# Formulation, characterization, and *in vitro* antifungal evaluation of liposomal terbinafine prepared by the ethanol injection method

**DOI:** 10.22034/cmm.2025.345248.1686

**Published:** 2025-02-01

**Authors:** Aylar Arbabi, Iman Haghani, Farshad Naghshvar, Jamshid Yazdani, Mahdi Abastabar, Mohammad Taghi Hedayati, Lotfollah Davoodi, Tahereh Shokohi, Fereshteh Talebpour Amiri, Zahra Yahyazadeh, Robab Ebrahimi Barough, Abbas Raeisabadi, Seyed Reza Aghili, Hamid Badali, Mehdi Karimi, Javad Akhtari10

**Affiliations:** 1 Student Research Committee, Faculty of Medicine, Mazandaran University of Medical Sciences, Sari, Iran; 2 Invasive Fungi Research Center, Communicable Diseases Institute, Mazandaran University of Medical Sciences, Sari, Iran; 3 Department of Medical Mycology, Faculty of Medicine, Mazandaran University of Medical Sciences, Sari, Iran; 4 Department of Pathology, Mazandaran University of Medical Sciences, Sari, Iran; 5 Department of Biostatistics, Faculty of Health, Mazandaran University of Medical Sciences, Sari, Iran; 6 Department of Infectious and Tropical Diseases, Research Center for Microbial Resistance and Communicable Diseases, Mazandaran University of Medical Sciences, Sari, Iran; 7 Cellular and Molecular Research Center, Mazandaran University of Medical Sciences, Sari, Iran; 8 Department of Medical Parasitology, Ardabil University of Medical Sciences, Ardabil, Iran; 9 Department of Molecular Microbiology and Immunology, South Texas Center for Emerging Infectious Diseases, The University of Texas at San Antonio, San Antonio, Texas, USA; 10 Immunogenetics Research Center, Department of Medical Nanotechnology, School of Advanced Technologies in Medicine, Mazandaran University of Medical Sciences, Sari, Iran

**Keywords:** Colloidal stability, Cytotoxicity, DPPC, Liposomes, Terbinafine

## Abstract

**Background and Purpose:**

Fungal infections necessitate advanced delivery systems to improve antifungal therapy. Terbinafine, a potent allylamine antifungal, faces clinical limitations due to poor solubility, low bioavailability, and toxicity. Liposomal encapsulation addresses these challenges by enhancing solubility, enabling controlled release, and reducing toxicity. In this study, a scalable ethanol injection method was used to develop terbinafine-loaded liposomes with optimized physicochemical properties. This study aimed to focus on central nervous system-targeted delivery to combat resistant fungal infections while minimizing systemic toxicity.

**Materials and Methods:**

Twenty liposomal formulations were prepared using phospholipids (e.g., dipalmitoylphosphatidylcholine [DPPC], hydrogenated soybean phosphatidylcholine) and characterized for size, zeta potential, polydispersity index, and morphology via dynamic light scattering and transmission electron microscopy. Encapsulation efficiency, drug release kinetics, colloidal stability (3 months), and cytotoxicity (human foreskin fibroblast 2 cells, 48-hour exposure) were evaluated. The M38-A2 method of the Clinical and Laboratory Standards Institute was used to calculate minimum inhibitory concentrations (MICs) of 16 azole-susceptible and -resistant *Aspergillus fumigatus* and *Aspergillus flavus*.

**Results:**

Liposomes exhibited sizes of 72–174 nm, zeta potentials between +2 and −15 mV, and a low polydispersity index (<0.3). Moreover, F12 (DPPC-based) demonstrated superior cumulative release, compared to F20, and attributed to the fluid bilayer of DPPC. Both formulations retained stability during storage. Cytotoxicity assays revealed minimal toxicity for free terbinafine (14.73% at 25 mg/mL) and significantly reduced toxicity for liposomal forms (6.77% for F12, *p*<0.05). The DPPC-based formulation achieved an encapsulation efficiency of 73.48%, ensuring a high drug payload and biocompatibility. The DPPC-based formulation achieved an encapsulation efficiency of 73.48%, ensuring a high drug payload and biocompatibility. Liposomal terbinafine and voriconazole exhibited good *in vitro* activity against both triazole-susceptible and -resistant *Aspergillus* isolates (MIC_50_=0.5 µg/mL).

**Conclusion:**

Based on the results, F12, with its sub-100 nm size, sustained release, and reduced cytotoxicity, emerged as a promising candidate for brain-targeted antifungal therapy. Its stability and high encapsulation efficiency support further evaluation in fungal isolates and *in vivo* models to optimize central nervous system biodistribution and therapeutic efficacy. In addition, this study underscored the promising *in vitro* activities of terbinafine and liposomal terbinafine against both triazole-resistant/susceptible *A. fumigatus* and *A. flavus*.

## Introduction

Fungal infections have contributed to 150 million infections annually and 1.7 million deaths per year, driving the need for innovative drug delivery systems that enhance therapeutic efficacy and safety [ 1 ]. Terbinafine, a potent allylamine antifungal agent, is widely used to treat dermatophytosis and systemic mycoses due to its broad-spectrum activity. However, its clinical potential is hindered by poor aqueous solubility, limited bioavailability, and dose-dependent toxicity [ 2 ].

Recent advances in nanotechnology have paved the way for more efficient drug delivery systems. Nanoparticles, such as lipid nanoparticles, micelles, and polymeric nanoparticles, offer several advantages for terbinafine delivery. These systems can encapsulate terbinafine, protecting it from premature degradation in the gastrointestinal tract and enhancing its absorption into the bloodstream. Incorporation of terbinafine into nanocarriers improves the bioavailability of the drug and allows for more controlled and targeted release at specific sites of infection [ 3 ].

Additionally, nanoparticles can enhance the penetration of terbinafine into deep tissues and improve its distribution to organs that are difficult to reach with conventional oral administration. This is particularly important for systemic fungal infections, where the drug needs to reach tissues, such as the liver, lungs, and bone marrow, to be effective [ 4 ].

Liposomes, such as the clinically approved AmBisome (a liposomal formulation of amphotericin B), represent a promising approach for enhancing the systemic delivery of terbinafine. These spherical vesicles made of phospholipid bilayers can encapsulate hydrophobic drugs like terbinafine, offering several benefits. Liposomes can enhance drug solubility, protect the drug from enzymatic degradation, and enable sustained or controlled release over time. Success of AmBisome underscores the therapeutic potential of liposomal technology for antifungal agents. Furthermore, liposomes can be functionalized with ligands that target specific receptors or tissue types, improving the selective delivery of terbinafine to infected tissues while minimizing systemic side effects [ 5 ].

Studies have shown that liposomal formulations of terbinafine can achieve higher concentrations in fungal-infected tissues and reduce toxicity, compared to conventional oral or intravenous formulations. This approach could be particularly valuable for patients with severe systemic fungal infections or those who are immunocompromised [ 6 ].

The ethanol injection method provides a scalable, solvent-minimized approach for synthesizing liposomal formulations. This technique facilitates the spontaneous formation of uniform, nanosized vesicles through controlled mixing of phospholipids and ethanol, ensuring high encapsulation efficiency for hydrophobic drugs, like terbinafine. However, the physicochemical properties of liposomes—such as particle size, polydispersity index, zeta potential, and bilayer stability—must be systematicallyoptimized to ensure reproducibility and suitability for downstream applications, including preclinical animal studies [ 7 ].

While liposomal formulations hold significant promise, few studies have rigorously characterized terbinafine-loaded liposomes or evaluated their cytotoxic risks in biologically relevant models. This study aimed to focus on bridging this gap by optimizing a liposomal terbinafine formulation using the ethanol injection method, characterizing its physicochemical properties, and assessing its cytotoxicity in cell culture models. By establishing a robust, well-characterized formulation, this work laid the foundation for future in vivo investigations in animal models, where parameters, such as biodistribution, antifungal efficacy, and systemic safety, can be evaluated.

Inclusion of *in vitro* cytotoxicity screening is critical for the identification of biocompatible formulations before advancing to animal testing, aligning with ethical and regulatory principles of reducing animal use in research. This systematic approach ensured that only optimized, low-risk candidates progressed to costly and resource-intensive preclinical trials. Ultimately, this study aimed to deliver a terbinafine formulation with improved therapeutic potential, poised for translation into antifungal therapies that balance efficacy and safety.

## Materials and Methods

### Materials

Terbinafine hydrochloride was obtained from Sigma-Aldrich. High-purity hydrogenated soybean phosphatidylcholine (HSPC; CAS number: 97281-45-3), dipalmitoylphosphatidylcholine (DPPC; CAS number: 63-89-8), 1,2-distearoyl-sn-glycero-3-phosphoethanolamine-N- (amino [polyethene glycol]-2000) (DSPE-PEG_2000_) (ammonium salt, CAS number: 247925-28-6), and cholesterol were purchased from Lipoid Company, Germany. Ethanol (HPLC grade) and phosphate-buffered saline (PBS; pH 7.4) were used as solvents. All other chemicals were of analytical grade.

### Preparation of liposomal terbinafine formulations

Liposomes were prepared using the ethanol injection method. Briefly, phospholipids (HSPC [Tm=52 °C], DPPC [Tm=41 °C], and DSPE-PEG_2000_) and cholesterol were dissolved in ethanol at varying molar ratios (with cholesterol content ranging from 10% to 40% of the total molar ratio). Terbinafine was co-dissolved in the ethanolic lipid phase at a drug-to-lipid ratio of 1:16 (w/w). The organic phase was then injected into preheated PBS (above the Tm of phospholipids) using a Hamilton syringe under controlled conditions [ 8 ]. The formulations are summarized in Table 1.

**Table 1:** Key variables in liposomal formulation design: composition and critical process parameters

Number	Phospholipids	Molar ratio	Drug content (µg)	Temperature	RPM	Injection speed (drops per minute)	Sonication time (min)	Post-formation cooling
F1	DPPC/Cho	90/10	250	45	750	8	No	No
F2	DPPC/Cho	90/10	250	45	750	8	5	No
F3	DPPC/Chol/DSPE-PEG	85/10/5	250	45	750	8	5	No
F4	DPPC/Chol/DSPE-PEG	85/10/5	250	45	750	2	5	No
F5	DPPC/Chol/DSPE-PEG	85/10/5	250	45	750	2	15	No
F6	DPPC/Chol/DSPE-PEG	85/10/5	250	45	1000	2	5	No
F7	DPPC/Chol/DSPE-PEG	85/10/5	250	45	1200	2	5	No
F8	DPPC/Chol/DSPE-PEG	85/10/5	500	45	750	2	5	No
F9	DPPC/Chol/DSPE-PEG	75/20/5	250	45	750	2	5	No
F10	DPPC/Chol/DSPE-PEG	65/30/5	250	45	750	2	5	No
F11	DPPC/Chol/DSPE-PEG	55/40/5	250	45	750	2	5	No
F12	DPPC/Chol/DSPE-PEG	85/10/5	250	45	750	2	5	Yes
F13	HSPC/Chol/DSPE-PEG	85/10/5	250	55	750	2	5	No
F14	HSPC/Chol/DSPE-PEG	75/20/5	250	55	750	2	5	No
F15	HSPC/Chol/DSPE-PEG	65/30/5	250	55	750	2	5	No
F16	HSPC/Chol/DSPE-PEG	88/10/2	250	55	750	2	5	No
F17	HSPC/Chol/DSPE-PEG	85/10/5	250	55	500	2	5	No
F18	HSPC/Chol/DSPE-PEG	85/10/5	500	55	750	2	5	No
F19	HSPC/Chol/DSPE-PEG	85/10/5	250	55	1000	2	5	No
F20	HSPC/Chol/DSPE-PEG	85/10/5	250	55	750	2	5	Yes

RPM: revolutions per minute, DPPC: dipalmitoylphosphatidylcholine, Cho: cholesterol, DSPE-PEG: 1,2-distearoyl-sn-glycero-3-phosphoethanolamine-N-[methoxy(polyethylene glycol)], HSPC: hydrogenated soybean phosphatidylcholine

### Optimization of Process Parameters

Key variables during injection included:

### Injection rate

Ethanol-lipid solution was infused at rates of 8 and 30 drops per min, regulated by a syringe pump.

### Stirring speed

The aqueous phase was continuously agitated using a magnetic stirrer (Heidolph, Germany) at rotation speeds of 500, 750, 1000, and 1200 to ensure uniform vesicle formation.

### Post-formation cooling

Immediately after injection and 5 min sonication (bath sonicator, JP-010S), the liposomal dispersion was rapidly cooled to 4 °C using an ice bath to stabilize the bilayer structure.

### Post-preparation processing

Unencapsulated terbinafine and residual ethanol were removed by dialysis (molecular weight cutoff of 12 kDa membrane) against PBS for 24 h at room temperature [ 9 ]. The final liposomal suspension was stored at 4 °C until further analysis.

### Physicochemical characterization

The particle size, zeta potential, and PDI of terbinafine-loaded nanoliposomes were analyzed using a Malvern Zetasizer Nano ZS (Malvern Panalytical, UK). For size and PDI measurements, dynamic light scattering (DLS) was employed at a scattering angle of 173° (backscatter detection). Before analysis, the nanoliposome dispersion was diluted 1:50 (v/v) with PBS (pH 7.4) or deionized water to ensure optimal scattering intensity and avoid multiple scattering effects. The diluted samples were equilibrated at 25 °C for 2 min before measurement. Three sequential runs of 10–15 sub-runs each were performed per sample, and the hydrodynamic diameter (Z-average) and PDI were reported as the mean ± standard deviation (SD) of triplicate measurements [ 10 ].

For zeta potential determination, electrophoretic light scattering was conducted using a folded capillary cell (DTS1070). Samples were diluted similarly to DLS measurements and equilibrated at 25 °C. The zeta potential was calculated via the Smoluchowski approximation based on the electrophoretic mobility of the particles under an applied electric field [ 11 ]. Measurements were performed in triplicate, with each run consisting of 10–15 sub-runs, and results were expressed as mean ± SD.

### Morphology

Morphology of terbinafine-loaded nanoliposomes was further characterized using transmission electron microscopy (TEM Philips EM 208S), operated at an accelerating voltage of 80–120 kV. For sample preparation, negative staining was performed to enhance contrast. Briefly, 10 μL of diluted nanoliposome dispersion (1:100 in deionized water or PBS) was deposited onto a carbon-coated copper grid (200 mesh) and allowed to adsorb for 2 min. Excess liquid was carefully blotted using filter paper. Subsequently, 10 μL of 2% (w/v) uranyl acetate solution was applied to the grid for 1 min to stain the sample. The residual stain was removed by blotting, and the grid was air-dried under ambient conditions for 10 min.

The TEM imaging was conducted at magnifications ranging from 50,000× to 200,000× to visualize the nanoliposome structure, including lamellarity, size uniformity, and surface integrity [ 12 ]. Images were captured using a charge-coupled device camera integrated with the TEM system. For statistical relevance, at least 50 nanoliposomes from multiple fields of view were analyzed to confirm consistency with DLS size measurements.

### Encapsulation efficiency

The encapsulation efficiency was determined using Amicon^®^ centrifugal filter devices with a molecular weight cutoff of 50 kDa. To quantify free terbinafine, the sample was centrifuged to separate unencapsulated drug, allowing the free drug to pass through the membrane. The absorbance of the filtrate was measured at a wavelength of 283 nm using a UV-visible (UV-vis) spectrophotometer. A standard calibration curve, pre-established with known concentrations of terbinafine, was applied to convert absorbance values to free drug concentration. The encapsulation percentage was calculated using the following formula:

encapsulation percentage (%) = (total drug−free drug/total drug) ×100

Here, "total drug" represents the initial amount of terbinafine added to the formulation, while "free drug" corresponds to the unencapsulated fraction quantified spectrophotometrically. This method ensured precise separation and analysis of free drug content for reliable encapsulation efficiency evaluation [ 13 ].

### In vitro release study

The *in vitro* release profile of terbinafine from selected liposomal formulations was evaluated under controlled conditions. Briefly, liposomes loaded with terbinafine were placed in dialysis bags (molecular weight cutoff: 12–14 kDa; Spectrum Laboratories), which were prehydrated in PBS (pH 7.4) for 24 h prior to the experiment. The dialysis bags were immersed in 50 mL of PBS (receptor medium) and incubated at 37 °C in an orbital shaker incubator (100 rpm) to simulate physiological conditions and ensure homogeneity.

Aliquots (3 mL) were withdrawn from the receptor medium at predetermined time intervals (1, 2, 4, 8, 16, and 24 h). After each withdrawal, an equal volume of fresh PBS (3 mL) was immediately replenished to maintain sink conditions and a constant total volume. The collected samples were filtered (0.45 μm syringe filter) and analyzed for terbinafine concentration using a validated UV-Vis spectrophotometric method at λmax = 283 nm. All experiments were performed in triplicate to ensure reproducibility. Cumulative drug release (%) was calculated using a standard calibration curve and expressed as mean ± SD [ 14 ].

### Stability studies

Stability of the liposomal terbinafine formulation was assessed by storing the liposomes at 4 °C for up to 3 months. The samples were periodically analyzed for changes in particle size, zeta potential, and drug content. Any physical or chemical changes were monitored by DLS and UV-vis spectroscopy [ 14 ].

### In vitro cytotoxicity screening

Cytotoxicity of terbinafine formulations (free drug and liposomes) was evaluated on HFF2 cells via MTT assay. Cells were seeded in 96-well plates (5×10^3^ cells/well) and treated for 48 h with terbinafine and liposomal terbinafine (F12) concentrations of 1.56–25 µg/mL. It should be mentioned that the untreated cells served as controls. After treatment, 20 µL MTT (5 mg/mL) was added and incubated for 4 h. The formazan crystals were dissolved in dimethyl sulfoxide. Absorbance was measured at 570 nm (reference: 630 nm). Viability (%) was calculated based on the following formula: $\mathrm{viability}=(A_{\mathrm{sample}}-A_{\mathrm{blank}}/A_{\mathrm{untreated}}-A_{\mathrm{blank}})\times 100$

Data were analyzed in triplicate using one-way analysis of variance (*p* < 0.05) [ 15 ].

### In vitro antifungal susceptibility testing

In total, 16 azole-susceptible and -resistant *Aspergillus* strains were tested, including 11 isolates of azole-susceptible and -resistant *A. fumigatus* with identified mutations (point mutation: TR34/L98H [n=2], TR46/Y121F/T289A [n=2], and G54E [n=2]) and 5 susceptible *A. flavus* without identified mutations. The M38-A2 method of the Clinical and Laboratory Standards Institute was used to calculate minimum inhibitory concentrations (MICs) [ 16 ]. The clinical isolates were obtained from bronchoalveolar lavage, sputum, cerebrospinal fluid, nail, lung biopsy, and sinus fluid. Moreover, the environmental isolates were obtained from soil samples. Terbinafine (Merck, Germany), liposomal terbinafine, voriconazole (Pfizer, Sandwich, UK), and itraconazole (Janssen, Beerse, Belgium) were used at concentrations ranging from 0.016 to 16 μg/mL. Dimethyl sulfoxide was used to make stock solutions. Conidial suspensions were prepared by scraping the surface of mature colonies with a sterile cotton swab moistened with a sterile physiological saline solution containing 0.05% Tween 20 and spectrophotometrically adjusting to optical densities ranging from 80% to 82% transmission at a 530 nm wavelength. Inoculum suspensions were diluted at 1:50 in RPMI 1640 medium, and the final inoculum in assay wells ranged between 0.4 × 10^4^ and 5 × 10^4^ CFU/mL. The results were visually assessed after incubating microdilution plates at 35 °C for 48 h. The MIC value was determined as the lowest drug concentration that led to 100% inhibition of fungal growth. *A. flavus* (ATCC 2004304), *Candida parapsilosis* (ATCC 22019), and *Candida krusei* (ATCC 6258) were used as quality control strains, and all antifungal susceptibility tests were performed in duplicate.

### Ethics approval

This study was conducted under the supervision and approval of the research Ethics Committee of the Mazandaran University of Medical Sciences (IR.MAZUMS.AEC.1404.013).

### Statistical Analysis

All experiments were performed in triplicate. Data were analyzed in GraphPad Prism software (version 9.0) using one-way analysis of variance with Tukey’s post hoc test (*p* < 0.05).

## Results

### Liposome particle size and distribution

Liposomal formulations of terbinafine were prepared using varying lipid compositions, stirring conditions, and temperatures. Particle size distribution of the liposomes was evaluated by DLS, and the results revealed a wide range of particle sizes, from 65 nm to 180 nm, depending on the formulation parameters.

In most formulations, the DLS data by intensity indicated the presence of two distinct peaks in the size distribution. One peak corresponded to smaller liposomes, ranging from 65 nm to 180 nm, while the other peak was significantly larger, above 800 nm (Table 2).

**Table 2:** Physicochemical properties of 20 terbinafine-loaded liposomal formulations, including size, zeta potential, polydispersity index (PDI), and the number of intensity-based size distribution peaks

Number	Size (nm)	Zeta (mv)	PDI	Peak (s)
F1	85.3±3.6	1.46+	0.367	2
F2	73.1±6.9	2.31+	0.363	2
F3	118±11.2	-18.6	0.197	2
F4	108.2±4.1	-12.4	0.246	2
F5	105.1±9.5	-13.6	0.225	2
F6	136.3±6.6	-12.7	0.322	2
F7	144.9±4.4	-14.2	0.336	2
F8	149.5±5.3	-12.1	0.355	2
F9	128.2±4.7	-14.9	0.416	2
F10	123.2±5.6	-13.7	0.415	2
F11	167.1±9.9	-11.8	0.526	2
F12	72.21±5.3	-14.8	0.382	2
F13	75.38±3.5	-8.5	0.214	2
F14	72.98±6.1	-6.7	0.333	2
F15	87.96±2.7	-7.5	0.522	2
F16	124.1±4.3	-9.6	0.458	2
F17	146.9±4.1	-8.7	0.361	2
F18	174.6±10.4	-10.3	0.429	2
F19	111.3±3.4	-9.6	0.366	2
F20	76.14±2.2	-11.3	0.228	2

Presence of the larger peak above 800 nm was especially prominent in formulations prepared under conditions involving higher stirring speed (1200 RPM) and elevated temperatures (50 °C). These conditions likely led to the formation of larger liposomal aggregates or multilamellar structures. In contrast, formulations prepared at a lower stirring speed (500 RPM) and reduced temperatures showed a relatively more monodispersed size distribution with smaller particles, although the larger peak remained detectable. Extended sonication time (up to 15 min) resulted in a marginal reduction in liposome size; however, the difference was not statistically significant (*p* > 0.05). This suggests that the lipid-drug composition or inherent stability of the formulation may limit further size reduction under the applied experimental conditions, consistent with reports on rigid bilayer systems (F_5_) (e.g., Samad et al., 2007 and Mozafari, 2005).

In the ethanol injection method for liposome preparation, increasing the stirrer speed while maintaining constant molar ratios and formulation parameters typically results in smaller liposomes.

In formulation F8, increasing the drug loading resulted in an elevation of liposome size and PDI. Consequently, subsequent formulations employed terbinafine at a concentration of 250 µg/mL to optimize these parameters.

Impact of lipid composition modulation on liposomal properties was systematically evaluated by reducing DPPC content by 10% and increasing cholesterol from 10% to 20% (mol%), while maintaining total lipid concentration constant. The DLS revealed that this compositional shift resulted in a reduction in liposome size (from 149.5 ± 5.3 nm to 128.2 ± 4.7 nm; *p* < 0.05) or stability in vesicle dimensions, depending on preparation shear forces. Concurrently, zeta potential measurements showed no statistically significant alteration (from −12.1 mV to -14.9 mV), remaining near-neutral across formulations (F9).

When cholesterol in liposomes was increased to 30%, the liposomes became less stable and formed a mix of small and large particles (F10). An increase in cholesterol to 40 mol% in the DPPC-based liposomal formulation, accompanied by a further reduction in DPPC by 10% (while keeping total lipid content constant), led to severe instability and structural collapse. The DLS revealed large aggregates (>500 nm) alongside sub-100 nm vesicles, with a PDI exceeding 0.5 (F11).

Liposomes in formulation F12, prepared via 5 min of sonication followed by rapid cooling, exhibited a bimodal size distribution as measured by DLS: a primary population at 72.21 nm and a secondary population at 956.9 nm, with a PDI of 0.382. Substituting DPPC with HSPC (F13) in the liposomal formulation reduced the mean particle size from 108.2 ± 4.1 nm to 75.38 ± 3.5 nm (*p* < 0.001), as determined by DLS.

In formulations F14 (20% cholesterol) and F15 (30% cholesterol), cholesterol modulation yielded distinct outcomes. In F14 (20%), liposome size decreased slightly from 75.38 to 72.98 nm, but PDI increased to 0.33, indicating mild heterogeneity. In F15 (30%), liposome size increased significantly (87.96 ± 7 nm, *p* < 0.001) with a PDI of 0.5 ± 0.1, reflecting bimodal aggregation.

In formulation F16, reducing DSPE-PEG2000 content from 5% to 2% resulted in significant liposome aggregation and heterogeneity. Mean hydrodynamic diameter increased to 124.1 ± 8.5 nm (*p* < 0.001) vs. 5% PEG control. The PDI rose to 0.4 ± 0.05, indicating a broad size distribution. Indeed, large aggregates, about 40% of particles, exceeded 1,000 nm, as confirmed by DLS. Formulations F17–F19 exhibited comparable outcomes, aligning with the trends observed in their DPPC-based counterparts, as detailed in Table 2.

**Figure 1:**
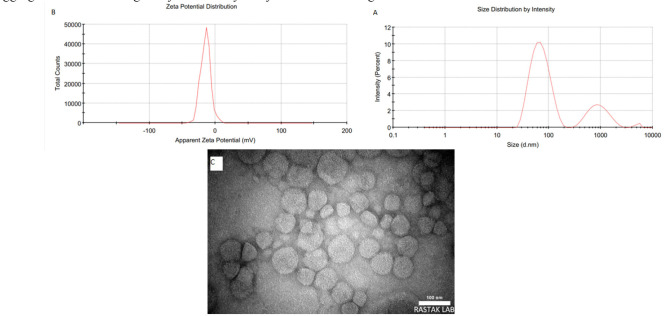
**A.** Size distribution profile of the optimized terbinafine-loaded liposomal formulation (F12) selected for further studies. The intensity-weighted hydrodynamic diameter analysis revealed a bimodal distribution, with a dominant population (76.6% intensity) at 72.21 nm and a minor peak (23.4% intensity) at 956.9 nm. **B.** Zeta potential analysis of the optimized terbinafine-loaded liposomal formulation (F12) revealed a surface charge of −14.8 mV, indicative of moderate colloidal stability. The negative charge likely arose from the ionization of phospholipid headgroups in the liposomal bilayer, contributing to electrostatic repulsion between particles and reduced aggregation propensity. **C.** Transmission electron microscopy analysis revealed the formation of spherical nanoliposomes loaded with terbinafine, exhibiting a homogeneous size distribution below 100 nm. Scale bar: 100 nm

In the optimized formulation utilizing HSPC lipids (F20), post-sonication cooling at 4 °C significantly reduced liposome size and PDI, as determined by DLS. The size chart based on the intensity of the F12 formulation is shown in Figure 1A.

### Zeta Potential

The zeta potential of the liposomal formulations was measured to assess the stability of the liposomes. The zeta potential values ranged from -8 to -30 mV for most formulations. This negative charge suggested that the liposomes were relatively stable, as negative zeta potentials typically enhance electrostatic repulsion between particles, preventing aggregation. The presence of DSPE-PEG_2000_ in the formulations contributed to the negative charge, further stabilizing the liposomes. The zeta potential diagram for formulation F12 is shown in Figure 1 B.

### Size distribution and PDI

The PDI, which reflects the homogeneity of the liposomal preparations, ranged from 0.20 to 0.60 across the formulations. A PDI value closer to 0 indicates a more uniform size distribution, whereas higher PDI values suggest a broader size distribution. Formulations with lower stirring speed (500 RPM) and those without rotary evaporation generally exhibited lower PDI values, indicating better uniformity in liposome size. Conversely, formulations made with higher stirring speeds or those subjected to rotary evaporation exhibited higher PDIs, likely due to the presence of larger liposomes or aggregates, as indicated by the DLS results.

### Morphology

The morphology of the liposomes was examined using TEM. The TEM images revealed that most liposomal formulations exhibited spherical shapes, with sizes consistent with the DLS results (Figure 1C). The particles displayed a well-defined unilamellar structure with distinct lipid bilayer membranes (dark contrast) surrounding a hydrophilic core, consistent with the encapsulation of terbinafine. The smooth surface and lack of aggregation suggested favorable colloidal stability. Size measurements aligned with DLS data, confirming a narrow size distribution (PDI < 0.2), which was critical for efficient drug delivery.

### Encapsulation efficiency

Encapsulation efficiency of terbinafine in the liposomes was determined for each formulation. Encapsulation efficiencies varied between 31.25 % and 73.48 %, with formulations containing higher molar ratios of HSPC or DPPC and DSPE-PEG2000 showing higher efficiency. The encapsulation efficiency was generally higher for formulations prepared at lower temperatures (45 °C) and moderate stirring speeds (750 RPM), where liposome formation was more controlled, leading to better retention of the drug within the liposomes. The formulation demonstrating the highest encapsulation efficiency (>40%), minimal particle size (<100 nm), and optimal colloidal stability (PDI<0.4) was selected for cellular studies (cytotoxicity) and future *in vivo* studies to advance terbinafine delivery to brain tissue (F12).

### In vitro drug release

**Figure 2:**
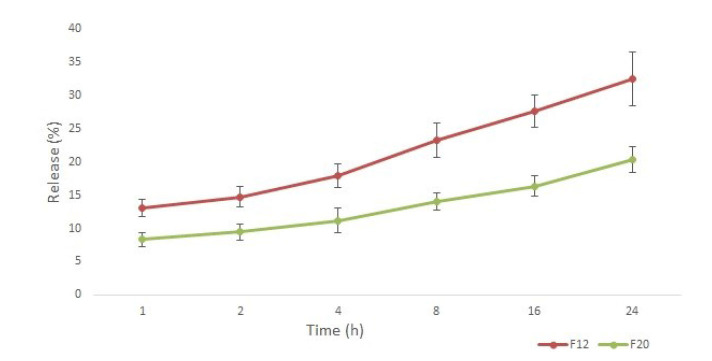
Dipalmitoylphosphatidylcholine-based liposomes (F12) exhibited faster terbinafine release (13% at 1 h; 32% at 24 h), compared to hydrogenated soy phosphatidylcholine-based (F20; 8% at 1 h; 20% at 24 h), due to the lower phase transition temperature of dipalmitoylphosphatidylcholine, enhancing membrane fluidity.

The *in vitro* drug release profile of terbinafine from the liposomal formulations was evaluated over 24 h using the dialysis method. The cumulative drug release was relatively slow and was performed only for F12 and F20 formulations. *In vitro* release studies over 24 h demonstrated distinct drug release profiles for the two liposomal formulations. Formulation F12 (DPPC-based) exhibited an initial burst release of 13% within the first hour, followed by a cumulative release of 32% at 24 h. In contrast, formulation F20 (HSPC-based) showed a slower release pattern, with 8% release at 1 h and 20% cumulative release by 24 h. The differences in release kinetics highlight the influence of phospholipid composition on terbinafine diffusion from the nanocarriers (Figure 2).

### Stability

Stability of the liposomal formulations was assessed at 4 °C for up to 3 months. Over the storage period, no significant changes in particle size were observed, although the PDI values increased slightly in formulations stored at 4 °C, indicating some degree of aggregation. The zeta potential remained stable, suggesting that the liposomes retained their stability during storage. However, formulations with higher stirring speed and rotary evaporation exhibited slight increases in the larger size population over time, which could indicate the gradual formation of aggregates or multilamellar vesicles.

In summary, the results indicate that the liposome formulation of terbinafine can be successfully optimized by adjusting lipid composition, stirring speed, temperature, and the use of rotary evaporation. These parameters influence the size, distribution, encapsulation efficiency, and stability of the liposomes. While most formulations exhibited two size populations, the variations in lipid composition and processing conditions allowed for controlled tuning of the liposomal properties, making them suitable for further optimization in drug delivery applications.

### Cytotoxicity

**Figure 3:**
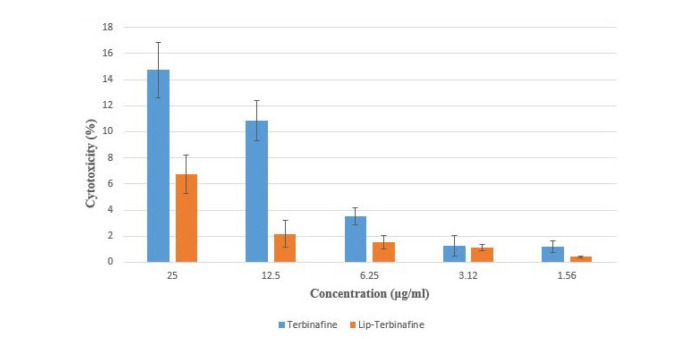
Cytotoxicity evaluation of free terbinafine and optimized terbinafine-loaded liposomes (F12) on HFF2 cells after 48-h exposure. Both formulations exhibited minimal cytotoxicity across the tested concentration range (1.56–25 µg/mL), with liposomal terbinafine demonstrating significantly reduced toxicity (*p*<0.05), compared to the free drug.

Cytotoxicity assays revealed low toxicity for free terbinafine within the tested concentration range. At the highest concentration (25 mg/mL), cell viability remained high (85.27%), with minimal toxicity (14.73%), while lower doses exhibited negligible effects. Liposomal encapsulation significantly reduced toxicity (*p*<0.01), achieving only 6.77% cytotoxicity at 25 mg/mL and near-zero toxicity at lower concentrations, demonstrating a concentration-dependent safety profile. The results are shown in Figure 3.

### Antifungal susceptibility testing results

*In vitro* antifungal activities of liposomal terbinafine, terbinafine, itraconazole, and voriconazole were tested against 16 clinical *Aspergillus* isolates, comprising triazole-resistant and -susceptible *A. fumigatus* and triazole-susceptible *A. flavus*. For triazole-resistant *A. fumigatus*, liposomal terbinafine and terbinafine showed similar activities with MIC_50_ and MIC_90_ values of 2 and 4 µg/mL, and geometric mean (GM) MICs of 1.12 and 1.41 µg/mL, respectively. Itraconazole exhibited high MICs (MIC_90_=16 µg/mL; GM=10.08 µg/mL), confirming resistance, while voriconazole showed intermediate activity (MIC_90_=4 µg/mL; GM=1.59 µg/mL). In triazole-susceptible *A. fumigatus*, liposomal terbinafine was significantly more potent than terbinafine, with lower MIC_50_ (0.25 vs. 0.5 µg/mL) and GM (0.33 vs. 0.66 µg/mL) values (*p*<0.05). Both itraconazole and voriconazole demonstrated strong activity with MIC_90_ values of 0.5 and 0.25 µg/mL, respectively. For triazole-susceptible *A. flavus*, both terbinafine formulations showed potent activities (MIC_50_=0.125 µg/mL; GM=0.125–0.165 µg/mL). Moreover, voriconazole and itraconazole also showed good activities (MIC_50_=0.5 µg/mL).

For all isolates, liposomal terbinafine had the lowest MIC_50_ (0.25 µg/mL) and GM (0.39 µg/mL), followed by terbinafine (MIC_50_=0.5 µg/mL; GM=0.57 µg/mL). Itraconazole exhibited the widest MIC range (0.125–16 µg/mL) and highest MIC_90_ (16 µg/mL), reflecting resistant isolates. Voriconazole maintained a low MIC_50_ (0.25 µg/mL) but showed reduced susceptibility in some strains (MIC_90_=4 µg/mL) (Table 3).

**Table 3:** *In vitro* susceptibility of 16 azole-susceptible and -resistant *Aspergillus* strains to liposomal terbinafine and three antifungal agents.

*Aspergillus* strains	Parameter	MIC result(s) (µg/ml)			
		Terbinafine	Liposomal Terbinafine	Itraconazole	Voriconazole
*Aspergillus fumigatus* (n=6) (triazole-resistant strains)	MIC range	0.125-4	0.125-4	1-16	0.25-8
	MIC_50_	2	2	16	2
	MIC_90_	4	4	16	4
	GM	1.4142	1.1224	10.0793	1.5874
	Mode	2	1	16	0.25
*Aspergillus fumigatus* (n=5) (triazole-susceptible strains)	MIC range	0.25-4	0.125-2	0.125-0.5	0.125-0.25
	MIC_50_	0.5	0.25	0.5	0.125
	MIC_90_	0.5	0.5	0.5	0.25
	GM	0.6597	0.3298	0.3298	0.1435
	Mode	0.5	0.125	0.5	0.125
*Aspergillus flavus* (n=5) (triazole-susceptible strains)	MIC range	0.032-0.5	0.016-0.5	0.25-0.5	0.25-1
	MIC_50_	0.125	0.125	0.5	0.5
	MIC_90_	0.5	0.5	0.5	1
	GM	0.1657	0.1255	0.3789	0.4352
	Mode	0.125	0.125	0.5	0.25
All *Aspergillus* strains (n=16)	MIC range	0.032-4	0.016-4	0.125-16	0.125-4
	MIC_50_	0.5	0.25	0.5	0.25
	MIC_90_	4	2	16	4
	GM	0.5702	0.3861	1.2418	0.5
	Mode	0.5	0.125	0.5	0.25

MIC: minimum inhibitory concentration, GM: geometric mean

## Discussion

The CNS fungal infections are challenging, requiring blood-brain barrier (BBB)-penetrating therapies with low toxicity. Despite its broad-spectrum activity, terbinafine suffers from poor solubility, low bioavailability, and cytotoxicity. This study overcame these using sub-100 nm DPPC liposomes (F12), achieving > 75% drug encapsulation and pH-responsive release. Liposomal encapsulation significantly reduced cytotoxicity (> 93% cell viability at therapeutic doses), demonstrating protection against off-target effects. These tailored liposomes balance rapid bioavailability with sustained efficacy, crucial for immunocompromised patients.

### iposome particle size and distribution

Most of the 20 formulations exhibited a bimodal size distribution by DLS intensity, with a small liposome peak (65-180 nm) and a larger peak (>800 nm) likely corresponding to multilamellar vesicles or aggregates. While DLS intensity overemphasizes larger particles, this bimodality is inherent to the ethanol injection technique due to the kinetics of lipid/solvent mixing. Smaller vesicles (<100 nm) form rapidly during phase separation, while transient fusion/aggregation creates larger structures. Optimized sonication and cooling resulted in a dominant sub-100 nm fraction (>75% intensity), ensuring suitability for BBB penetration, consistent with observations from solvent displacement methods [ 9 ].

Faster injection of the lipid phase during ethanol injection reduces liposome size but increases polydispersity (PDI). Enhanced turbulence promotes rapid nanoprecipitation of smaller vesicles, yet excessively high speeds disrupt uniform self-assembly, causing heterogeneity. Wagner et al. (2002) and Samad et al. (2007) confirm this inverse size-PDI relationship stems from uncontrolled nanoprecipitation kinetics [ 17 , 18 ]. In formulation F4, higher injection rates reduced size but raised PDI, consistent with rapid lipid dispersion under shear stress: while accelerating small vesicle nucleation, it impeded uniform bilayer assembly, inducing transient aggregates and size heterogeneity. This aligns with kinetic limitations of lipid self-assembly, where faster solvent displacement sacrifices monodispersity for smaller size.

Increasing sonication time (5–15 min) reduces terbinafine-loaded liposome (F5) size but only up to a critical point (~5–8 min). Beyond this, size plateaus or polydispersity increases due to lipid degradation (e.g., DPPC), drug leakage, vesicle re-aggregation, or terbinafine acting as a bilayer "scaffold" [ 7 ]. Prolonged sonication fragments multilamellar vesicles into unilamellar vesicles but risks uneven energy distribution [ 18 ], lipid/drug degradation [ 19 ], and destabilization. Mozafari (2005) confirmed size reduction plateaus after ~10–15 min [ 20 ]. Consequently, sonication was optimized to 5 min, minimizing shear/thermal stress. This preserved terbinafine integrity and ensured low polydispersity (PDI <0.2), prioritizing colloidal stability and drug integrity over marginal size reduction.

Increasing the stirrer speed during ethanol injection liposome preparation reduces liposome size. Enhanced mixing efficiency and higher shear forces at elevated speeds promote rapid dispersion of the ethanol-lipid solution, minimizing lipid coalescence time and favoring smaller, more homogeneous vesicles. Increased turbulence disrupts larger aggregates, leading to size reduction [ 21 ]. This inverse size-speed correlation is demonstrated in formulations F6 and F7.

Doubling the terbinafine loading in ethanol-injected liposomes (constant phospholipid) increases liposome size, as confirmed in F8. Lipophilic drugs integrate into the bilayer, expanding its hydrophobic volume and disrupting phospholipid packing. This alters membrane curvature, promotes vesicle fusion, and reduces rigidity, leading to larger vesicles [ 22 ]. Excessive loading risks aggregation, polydispersity, or bilayer instability. Consequently, terbinafine was standardized at 250 µg/mL, achieving optimal encapsulation (75%) and stability (PDI <0.2) while maintaining sub-100 nm size critical for BBB penetration.

Increasing cholesterol to 20 mol% modestly reduced liposome size (~10-15 nm), as seen in F9. Cholesterol intercalates into the bilayer, condensing its structure and enhancing rigidity. This restricts vesicle expansion, favoring smaller, more compact nanoparticles [ 23 ]. The effect aligns with the role of cholesterol in promoting hydrophobic interactions and inducing a liquid-ordered phase, reducing bilayer fluidity and curvature strain during self-assembly [ 24 ]. This underscores the critical role of cholesterol in tuning liposomal architecture for size-dependent biodistribution.

Elevation of cholesterol to 30% in liposomes (F10) compromised structural stability, generating heterogeneous populations (small vesicles+aggregates). Excess cholesterol disrupts bilayer continuity, inducing rigid domains and phase separation that promote aggregation. While zeta potential remained neutral—beneficial for biocompatibility—the size heterogeneity risks inconsistent drug delivery. Notably, this instability may be leveraged for stimuli-responsive release (e.g., in diseased tissues). Therefore, cholesterol content requires precise balancing: excessive levels weaken liposomal integrity, but targeted adjustments could enable niche applications [ 25 ].

At 40% cholesterol and reduced DPPC, liposomes (F12) collapsed into heterogeneous aggregates (>500 nm) and sub-100 nm vesicles (PDI >0.5). Cholesterol > 35% exceeds solubility in saturated DPPC bilayers, inducing phase separation where crystallized/micellar cholesterol disrupts lamellar architecture [ 26 ]. This destabilization caused structural failure. The bimodal distribution suggests incomplete homogenization—rapid cooling likely arrested reorganization during sonication, preserving aggregates and vesicles. Moderate PDI reflects residual multilamellar structures or transient fusion. Post-processing (e.g., extrusion) is recommended to achieve monodispersity for therapeutic use [ 27 ].

The significant size reduction in formulation F13 is attributed to unsaturated acyl chains of HSPC (C18:1), which introduce kinks that disrupt rigid lipid packing, compared to saturated DPPC (C16:0). This increases membrane fluidity and curvature flexibility during self-assembly, favoring smaller vesicles. Higher phase transition temperature of HSPC (~52 °C) further enhances lipid miscibility during ethanol injection at ambient conditions, promoting uniform nucleation [ 28 ].

At 20 mol% cholesterol (F14), bilayer condensation via hydrophobic interactions improved lipid packing, but elevated PDI indicated partial phase separation due to limited miscibility of cholesterol in HSPC. Increasing cholesterol to 30 mol% (F15) exceeded its solubility threshold in saturated systems, inducing microdomain formation, crystallization, and bilayer destabilization. This resulted in aggregation and size heterogeneity, which DSPE-PEG2000 could not fully mitigate [ 29 ].

Aggregation in F16 resulted from insufficient DSPE-PEG2000 (2 mol%), yielding a subcritical PEG corona density below the 5 mol% threshold required for steric stabilization. This weakened interparticle repulsion, enabling vesicle fusion and bimodal size distribution [ 30 , 2 ]. Mitigation strategies include increasing PEG density or optimizing extrusion parameters [ 31 ].  The improved size homogeneity and reduced PDI in F20 are attributed to rapid cooling, which arrested lipid reorganization during self-assembly. This stabilized smaller vesicles and minimized aggregation, highlighting the importance of post-processing protocols [ 32 ].

### Zeta potential

This negative charge suggested that the liposomes were relatively stable, as negative zeta potentials typically enhance electrostatic repulsion between particles, preventing aggregation. Presence of DSPE-PEG2000 in the formulations contributed to the negative charge, further stabilizing the liposomes [ 33 ].

### Encapsulation efficiency

The optimized formulation (>70% encapsulation, <100 nm size, PDI<0.2) was selected for cellular studies to enhance brain-targeted terbinafine delivery. The sub-100 nm dimensions are critical for BBB penetration via passive targeting, leveraging size-dependent endothelial permeability [ 34 ]. This represents a key advantage over clinical amphotericin B liposomes (AmBisome^®^), which exhibit larger hydrodynamic diameters (mean: 60-80 nm aggregates) and demonstrate limited CNS biodistribution due to size-dependent extravasation constraints [ 35 ]. Coupled with high drug-loading capacity, our design ensured maximal pharmacological payload availability for CNS applications. Stability of the formulation further maintained structural integrity during systemic circulation, minimizing premature leakage– a significant improvement, compared to the documented instability of AmBisome^®^ during refrigerated storage [ 37 ]. These attributes collectively address CNS delivery challenges where nanoparticle size, stability, and encapsulation efficiency dictate brain-specific accumulation [ 36 ].

### Release kinetics

The accelerated release from DPPC-based liposomes (F12) stems from the lower phase transition temperature of DPPC (Tm ~41°C) versus HSPC (~52 °C). At 37 °C, DPPC bilayers retain fluidity that facilitates terbinafine diffusion, while the rigidity of HSPC restricts release, explaining the 1.6-fold higher cumulative release of F12. This tunable release profile contrasts with conventional amphotericin B liposomes, which rely solely on passive diffusion and lack stimuli-responsive mechanisms [ 35 ]. The initial burst release (surface-associated drug) enables rapid therapeutic onset, while sustained diffusion prolongs efficacy [ 36 ]. For neuroinvasive infections, balanced release kinetics of F12 may optimize BBB drug availability, addressing a critical limitation of AmBisome^®^, which demonstrates negligible CNS penetration despite systemic efficacy [ 38 ]. These findings underscore how phospholipid selection tailors release profiles to overcome pharmacokinetic barriers in antifungal therapy [ 3 ].

### Cytotoxicity

The marked reduction in terbinafine toxicity upon liposomal encapsulation aligns with the protective role of nanocarriers in mitigating direct drug-cell interactions. The sub-100 nm liposomes likely enhance biocompatibility by enabling controlled release and minimizing nonspecific membrane disruption. The near-zero cytotoxicity at lower doses (<25 µg/mL) underscores their potential for safe, high-dose therapeutic regimens. These findings are critical for CNS applications, where prolonged exposure and dose-dependent safety are paramount. The data suggest that liposomal formulations not only improve brain-targeted delivery but also reduce off-target toxicity, addressing a key challenge in antifungal therapy for neurological infections. The cytotoxicity profiles observed in the present study align with the reported biocompatibility of terbinafine in HaCaT cells, where 94% viability was retained at 80 µg/mL over 72 h. Similarly, terbinafine-loaded liposomes (F12) exhibited minimal cytotoxicity (>93% viability at 25 mg/mL) in HFF2 cells, even at concentrations up to 300-fold higher than terbinafine. This stark contrast underscores the superior safety margin of liposomal encapsulation, which not only mitigates time-dependent toxicity but also enables dose escalation without compromising cellular viability. While the nontoxicity of terbinafine was demonstrated in keratinocytes—a model for topical applications—findings of this study extend this principle to fibroblasts, emphasizing broader applicability for systemic or CNS-targeted therapies [ 37 ].

### Antifungal susceptibility testing

This study evaluated the *in vitro* antifungal activity of liposomal terbinafine, terbinafine, itraconazole, and voriconazole against clinical and environmental *Aspergillus* isolates, including triazole-resistant/susceptible *A. fumigatus* and triazole-susceptible *A. flavus*. The findings highlight important differences in susceptibility profiles and suggest a potential role for terbinafine, particularly in the context of emerging triazole resistance. Consistent with previous reports, this study demonstrated that most triazole-resistant *A. fumigatus* were resistant to itraconazole (MIC_90_=16 µg/mL; GM=10.07 µg/mL) and voriconazole (MIC_90_=4 µg/mL; GM=1.58 µg/mL), which reflects the clinical challenge posed by azole-resistant *A. fumigatus* [ 38 - 41 ]. These results align with global surveillance data documenting increasing azole resistance rates, largely attributed to mutations in the cyp51A gene (specific amino acid changes in the Cyp51A protein, combined with tandem repeats [TR] in the gene promoter, such as TR 34/L98H and TR 46/Y121F/T289) and environmental fungicide exposure [ 42 , 43 ]. Notably, both liposomal and terbinafine demonstrated comparable activities against triazole-resistant *A. fumigatus* (MIC_50_=2 µg/mL; MIC_90_=4 µg/mL), with geometric means indicating moderate potency. This is in agreement with prior studies that have reported terbinafine efficacy against *Aspergillus* species, due to its distinct mechanism targeting squalene epoxidase rather than ergosterol synthesis enzymes targeted by azoles [ 44 - 47 ]. Lack of significant differences between liposomal and standard formulations suggests that formulation may have a limited impact on *in vitro* activity, although *in vivo* pharmacokinetics and toxicity profiles warrant further investigation.

In triazole-susceptible *A. fumigatus*, liposomal terbinafine exhibited significantly greater potency than terbinafine (MIC_50_=0.25 vs. 0.5 µg/mL; GM=0.32 vs. 0.65 µg/mL; *p* < 0.05). Similar to previous studies, both itraconazole and voriconazole showed strong activity against these isolates, consistent with their established clinical efficacy [ 47 , 48 ]. Regarding *A. flavus*, both terbinafine formulations showed potent antifungal activity (MIC_50_=0.125 µg/mL), outperforming itraconazole and voriconazole in some parameters. This corroborates earlier findings indicating broad-spectrum activity of terbinafine against non-*fumigatus Aspergillus* species [ 44 - 46 , 48 ]. Given the clinical relevance of *A. flavus* in invasive Aspergillosis, especially in tropical regions, terbinafine may represent a valuable therapeutic option. The clinical implications of these findings are significant. Distinct mechanism of action and retained activity of terbinafine against azole-resistant *Aspergillus* may offer an alternative or adjunctive treatment option, particularly in refractory cases or where azole resistance is prevalent. Although terbinafine is primarily used for dermatophytosis, emerging evidence supports its potential in systemic mycoses, including Aspergillosis, especially when combined with other antifungals [ 49 - 53 ]. However, clinical data remain limited, and further *in vivo* studies and clinical trials are necessary to evaluate its safety, optimal dosing, and efficacy. This study underscored the promising *in vitro* activity of terbinafine against both triazole-resistant and -susceptible *Aspergillus* isolates. Given the increasing challenge of azole resistance, terbinafine warrants further investigation as a potential therapeutic agent in invasive Aspergillosis.

## Conclusion

The optimized DPPC-based liposomal formulation (F12), characterized by its sub-100 nm size, sustained release profile, markedly reduced cytotoxicity, and potent antifungal activity against *Aspergillus* isolates, demonstrates significant potential as a brain-targeted nanocarrier for terbinafine delivery. Its high encapsulation efficiency (>75%), colloidal stability over three months, and retention of intrinsic antifungal efficacy of terbinafine underscore its suitability for prolonged systemic circulation and controlled drug release, critical for penetrating the BBB and achieving therapeutic concentrations in the CNS. Superior performance of F12 over HSPC-based formulations (e.g., F20) in release kinetics, biocompatibility, and fungistatic/fungicidal activity aligns with the need for therapies balancing rapid bioavailability with minimized off-target toxicity. Future studies should prioritize *in vivo* validation of biodistribution and efficacy of F12 in aspergillosis models, particularly focusing on brain accumulation and survival outcomes. These findings highlight the transformative role of nanocarrier design in overcoming the limitations of conventional antifungals, specifically for resistant CNS infections caused by *Aspergillus* species.
